# Closing the GAP in Antimicrobial Resistance Policy in Benin and Burkina Faso

**DOI:** 10.1128/msystems.00150-22

**Published:** 2022-07-27

**Authors:** Salla Sariola, Andrea Butcher, Jose A. Cañada, Mariette Aïkpé, Adélaïde Compaore

**Affiliations:** a Department of Sociology, University of Helsinki, Helsinki, Finland; b Department of Sociology, Philosophy and Anthropology, University of Exeter, Exeter, United Kingdom; c Laboratory of Applied Medical Anthropology, University of Abomey-Calavi, Abomey-Calavi, Benin; d Clinical Research Unit of Nanoro, Institut de Recherche en Sciences de la Santé, Nanoro, Burkina Faso; Institute of Urban Environment, Chinese Academy of Sciences

**Keywords:** antimicrobial resistance, Benin, Burkina Faso, West Africa, inequality, low-income countries, qualitative research

## Abstract

Antimicrobial resistance (AMR) is a global concern that is geographically unevenly distributed, with low- and middle-income countries and African countries suffering in particular. The World Health Organization (WHO) Global Action Plan (GAP) for antimicrobial resistance identified five key objectives that aim to ensure the continued treatment and prevention of infectious diseases with the use of antibiotics. Countries signatory to the WHO GAP are expected to develop their own national action plans (NAPs) based on the global model. How low-income countries are able to achieve the GAP objectives is not well understood. This paper analyzes the progress of two West African countries in achieving the GAP targets, Benin and Burkina Faso, countries among the lowest bracket in the World Development Index. We present qualitative data from interviews and focus group discussions with local policy-makers, nurses, doctors, animal breeders, veterinarians, and laboratory researchers, supported by participant observations and surveys within rural communities. The analysis is organized according to the five WHO GAP objectives to show the countries’ challenges in fulfilling them. The analysis shows that there are shortcomings in all of the WHO GAP areas in the two countries, making it a compounded and multifactorial problem—a stacking of lacks. In such contexts, calibrating a society toward AMR resilience/prevention requires overall development and attention to interdependencies. Active local research and policy communities with international, sustained financial support are essential for achieving the targets.

**IMPORTANCE** The burden of antimicrobial resistance (AMR) is unequally distributed across the globe. Low-income countries face a more severe AMR situation and have fewer means to solve the problem. This paper brings out the voices of local experts, policy-makers, and members of the community in Benin and Burkina Faso across human health, animal health, and food production sectors, where the majority of antibiotic use is concentrated. We describe the difficulties that they face in implementing global action plans, targets set by the World Health Organization, for securing antibiotics and preventing the spread of antimicrobial resistance. This paper argues that the various deficits in implementation are stacked, multisectoral, and compounded. We highlight the role of active local scientists and policy-maker networks in setting priorities to address the AMR problem; however, their activities need technical and financial support from international partners.

## INTRODUCTION

Antimicrobial resistance (AMR) is an enormous global concern that has far-reaching implications for various societal sectors. The risks of AMR are geographically unevenly distributed, with low- and middle-income countries and African countries suffering in particular. The World Health Organization (WHO) Global Action Plan (GAP) for antimicrobial resistance has identified five key objectives with the aim of ensuring the continued treatment and prevention of infectious diseases with the use of antibiotics. The five WHO GAP objectives are to (i) improve awareness of the resistance problem, (ii) strengthen research and surveillance, (iii) reduce incidence by improving sanitation, (iv) optimize the use of antibiotics in humans and animals, and (v) develop the economic case for alternatives. The GAP was established in 2015, and countries signatory to the WHO are expected to develop their own national action plans (NAPs) based on the global model. So far, 149 countries have finalized their NAPs (https://www.who.int/activities/supporting-countries-with-national-action-plan-implementation; https://www.who.int/teams/surveillance-prevention-control-AMR/national-action-plan-monitoring-evaluation/library-of-national-action-plans). As such, the GAP is a strong framework of standardized guidance on what can be done to prevent the onset and spread of AMR and to secure the reliability of existing antibiotics. A key characteristic of the GAP is that it is multisectoral; it straddles three different domains under a One Health umbrella: humans, animals, and the environment.

This paper focuses on the attempts to achieve the GAP objectives in two West African countries, Benin and Burkina Faso (BF). There are shortcomings in all of the areas, and attention to the GAP objectives reveals the interdependence that exists across them, making AMR prevention a compounded and multifactorial problem—a stacking of lacks. These countries are among the lowest bracket in the World Development Index, and according to the Global Health Security Index, Burkina Faso and Benin are among the least prepared, ranking 145th and 150th out of 195 countries, respectively. Both countries lack robust surveillance and evaluation mechanisms and have not participated in the WHO Global Antimicrobial Resistance and Use Surveillance System (GLASS) database. Sanitation and health care infrastructures, where they exist, are poorly developed. According to the Centers for Disease Control and Prevention (CDC), the leading causes of death in both countries are neonatal disorders, malaria, respiratory infections, and diarrheal diseases that are directly relevant to AMR. Both countries are members of the West African Economic and Monetary Union (Union Économique et Monétaire Ouest-Africaine [UEMOA]), a customs union of eight Francophone West African states. Member states are bound by union legislation governing the importation, distribution, and sale of clinical and veterinary medicines. In practice, antibiotics can be purchased without a prescription for both human and animal use, although the informal sale of pharmaceuticals has been prohibited in Benin since February 2017. Neither government has verified their NAP, but there are ongoing efforts to translate these into practice. Burkina Faso’s NAP is listed on the WHO NAP website, while Benin’s is not. Systematic data about AMR prevalence do not exist at the country level, but worrying levels of AMR have been found in clinical, animal, and environmental samples in both countries: blood samples from children (Benin) (1); blood, fecal, and urine samples from children (BF) (2); gonorrhea, especially among communities of sex workers and men who have sex with men (MSM) (BF) (3); chickens and guinea fowl sold at markets for consumption (BF) (4); irrigation water and the lettuce (BF) (5); and across drinking water, groundwater, and hospital wastewater (Benin) (6). Multidrug resistance was common across them.

In this paper, we present qualitative data from interviews and focus group discussions with local policy-makers, nurses, doctors, animal breeders, veterinarians, and laboratory researchers, supported by participant observations and surveys within rural communities in Burkina Faso and Benin to obtain their perspective on where their countries are with the targets of the GAP policy. We have organized the five key WHO GAP objectives as the thematic logic with which our empirical material is presented and show the challenges in fulfilling these objectives owing to interdependencies.

Our analysis shows that tackling the problem of AMR spread is not something that can be resolved by addressing one societal domain or with one particular “technofix.” Calibration of a society toward AMR resilience/prevention requires overall development. It requires addressing the various lacks and attention to the ways in which the lacks interact with each other. We argue that frameworks are not enough to reach the aim, but concerted attention to the different starting points within which countries are working to implement their national plans is needed. Social scientific research and interdisciplinary research with local policy-makers are required to identify priority areas that are locally relevant.

## RESULTS

### GAP objective 1: improving awareness of AMR through training and education.

The GAP sets out raising awareness as the key starting point for ensuring continued success in antibiotic treatment. The general sense among policy-makers and experts across the two countries was that AMR awareness among members of the lay public regarding antimicrobial resistance was low. A medical doctor in Burkina Faso described the level of public awareness of AMR as “very low, very low, very low, very low. It’s very low.”

In contrast, those who relied on antibiotics for their profession or occupation, such as pharmacists, breeders, farm managers, veterinarians, doctors, and nurses, etc., disputed that there was a lack of awareness about the need to reduce the use of antibiotics. Yet on many occasions, they continued to prescribe and use antibiotics contrary to their best knowledge. In the process of deciding whether to treat a patient or an animal with antibiotics or not, prescribers described negotiations between the patient at hand (human or animal) and broader socioeconomic concerns. A veterinarian from Benin in a focus group explained awareness about antibiotics among veterinarians as follows:

I believe that the problem does not arise much in terms of training. The training that we have received, we know all that. We cannot say that there is a knowledge gap in relation to this problem. The problem is much more at the level of animal carers. While many breeders understand today about AMR, we don’t have any specific diagnostic elements to support their work. When there is a problem, we should be able to make a precise diagnosis to know what the animal is suffering from and the antibiotic that would work for this pathology. At given moments, we act, we use this, it doesn’t work, we go further, it doesn’t work. There are some breeders who start directly with the drugs, and when they fail, they call you, and now you have trouble knowing what you are going to prescribe to them because if you want to kill a fly and you use a gun or an axe and it fails, it is really complicated to go back again. So we don’t have a training problem, I want to clarify; all those who are professional know the problem. Maybe there is a problem of information at the level of the practitioners, at the level of the breeders, of the producers.

The scenario that this veterinarian describes is typical of our data, in which it is not the absence of knowledge but the absence of access to essential infrastructure that allows them to observe good antimicrobial practice, for example, diagnostics, that is driving the use of antibiotics. This first example of various lacks begins to unravel our argument about the stacked shortcomings to curb AMR and that the five GAP objectives are interconnected. This example points to the precarity for animal breeders whose livelihoods depend on rearing healthy animals and how circumstances drive them to “kill a fly with a gun” to secure their flock. This vignette also shows how the lack of regulation of the sale of antibiotics is what enables their easy accessibility. A medical doctor attributed the problem of widespread antibiotic use not to a lack of awareness but to the lack of regulation of sales and pressures on health care practitioners to deliver treatment:

I’m not sure that it is a problem of awareness. Pharmacists know the importance of antibiotics and having a prescription. I think that it is maybe a lack of our policies; you know, here, the physician delegates more tasks to the nurse. In some countries, only physicians prescribe antibiotics, but in our context, everyone in health organizations can prescribe. It’s legal but there is no control. They do it because they want. It’s also an economical determinant.

Our findings suggest that the idea that individual behaviors would change as a result of increased awareness will not deliver the most effective results in reducing AMR. This finding has considerable support from the literature where several reviews (7, 8) on the (long-term) impacts of antibiotic awareness and individual behavior change programs suggest that increasing knowledge is not effective without considering other determining factors. Understanding and addressing the structures that shape why and how people prescribe and use antibiotics in the way that they do, including economic pressures and a lack of access to diagnostics and health care, etc., are crucial.

### GAP objective 2: strengthening research and surveillance.

The WHO guidance for NAPs emphasizes the roles of research and surveillance as essential components for generating knowledge of the AMR situation and emphasizes the need for collaboration across disciplines, including the social sciences, for understanding the mechanisms, development, and spread of AMR and to create novel interventions and alternatives to antibiotics. Various societal bodies are highlighted as being relevant: national governments, intergovernmental organizations, agencies, professional organizations, nongovernmental organizations (NGOs), industry, and academia. Surveillance of AMR should generate information on “the incidence, prevalence, range across pathogens, and geographical patterns related to antimicrobial resistance to guide the treatment of patients; to inform local, national, and regional actions; and to monitor the effectiveness of interventions” and “how resistance develops and spreads within and between humans and animals and through food, water, and the environment (https://www.who.int/publications/i/item/9789241509763).” Surveillance is an important tool for optimizing antibiotic use in clinical and veterinary decision-making. Having surveillance data available, however, assumes a surveillance system that hinges on the existence of various tools and infrastructure for testing, sample transport, equipment, programs, streamlined protocols, platforms, and indicators of analysis, etc.

In terms of the implementation of surveillance, our interlocutors in West Africa were highly motivated researchers and health practitioners interested in participating in international research networks and training events and worked tirelessly to equip their laboratories and train their research staff to be able to fill data gaps from their countries (9), all of this despite the lack of resources and initiative from institutional bodies responsible for the implementation of the NAP. Nationwide surveillance networks nonetheless remained lagging, which reflects the status of diagnostic infrastructures and capacities across different infectious diseases across West Africa (10–12).

The head of the national veterinary laboratory in Burkina Faso explained that they did not have a standardized system for surveillance data acquisition, they had no infrastructure for sample transportation, and they receive data for only a small portion of the tests done because “it’s not in the vets’ daily routine.” The data that they receive come from veterinarians or paraveterinarians who live nearby and who are committed. This was due to a lack of resources: shipping samples from outside the capital to where the national veterinary laboratory is located is expensive, and no expense compensation is provided. The head of the laboratory described surveillance as follows:

A: We have two types of surveillance: active surveillance and passive surveillance.

Q: What?

A: Passive. Passive surveillance. In passive surveillance, we are just waiting and receiving all the samples from the field. We don’t do anything (collect, initiate), we’re just here and wait, and when it comes, it is positive or negative. But in active surveillance, we would make our own protocol of surveillance, and this is how we would know the prevalence of disease. But active surveillance needs money. You have to transport, you have to pay agents, all of them. So, we don’t do this…

Q: You haven’t really done any active surveillance…?

A: For some diseases, we do it. For projects or something that we have some resources for, we do it. But it is not our routine activity. It is an exception. It would be better for us to do more because without it, we don’t know the real situation in our country. It is a challenge.

Similarly, in Benin, our interviewees described how laboratories have considerably improved in the past decade. However, as an AMR policy-maker from Burkina Faso explained, what also does not exist yet is a surveillance system:

It doesn’t exist. There are *ad hoc* studies on the prevalence of bacterial agents, done in a piecemeal fashion at a given level. So there is no national data that we can collect to disseminate. We don’t have reliable data, coming from the base of the laboratories, because we did not harmonize surveillance systems. Then, surveillance being a system of systematic uninterrupted data collection and their subsequent analysis, by this definition, we are not in surveillance yet…

This policy-maker talks about the reality where surveillance ends up depending on individual studies carried out in specific laboratories by specific individuals (most often) participating in international collaborations. While efforts have been made to improve surveillance at national levels, trained by different international partners, different laboratories have very different working conditions and foci, making them unable to implement what they have learned in a standard fashion. Presently, while surveillance has not yet reached a concerted effort, it is important to mention that Burkina Faso has an important number of professionals and laboratories involved in its surveillance network, and the building of Benin’s network is ongoing.

### GAP objective 3: reducing incidence through improving sanitation, hygiene, and infection prevention measures.

The third objective of the GAP is perhaps the most ambitious and fraught in the two countries. A call for better hygiene and infection prevention measures is essential to limit the development and spread of antimicrobial-resistant infections, which requires attention to improve the management of hygiene, especially in rural areas. However, reducing the incidence of AMR is closely connected to other lacks, diagnostics, regulation, and financial protections, etc. The GAP addresses only improving sanitation, vaccinations, and human-animal relationships, which is what we address in this section. However, concrete interventions that would also support the implementation of this objective would include, for example, improving biosecurity for farmers, preventing hospital infection by sanitation, and treating hospital and other wastewaters.

In 2016, according to World Bank (WB) development indicator statistics, the mortality rates attributed to unsafe water, unsafe sanitation, and a lack of hygiene were 59.7% (Benin) and 49.6% (Burkina Faso). In the two countries, open sewage is the norm in cities, and only 16% (Benin) and 19% (Burkina Faso) of the population have basic sanitation services, that is, toilets that are not shared with other households and include flush/pour flush to piped sewer systems, septic tanks, or pit latrines; improved ventilated pit latrines; composting toilets; or pit latrines with slabs. Based on our findings, open defecation is common in over 60% of households in rural Burkina Faso. In rural areas, villages share toilets or have designated places in the fields and forests where people defecate. These areas are roamed by domestic animals and do not usually have washing facilities available, which poses a risk for the spread of infections. The percentages of people living in households that have a handwashing facility with soap and water available on the premises are 10% (Benin) and 11% (Burkina Faso). Handwashing facilities here mean a sink with tap water, buckets with taps, tippy-taps, and jugs or basins designated for handwashing.

According to our interviews, people understood the significance of clean water for good health. However, they had trouble accessing water and spent considerable time and money daily accessing clean water. Alternatively, they simply did not drink, leading to dehydration. In the capital cities of both countries, it was possible for households to connect to urban water supply networks or drill a private borehole and install a water tank. Poorer households had the option of using wells, boreholes, river water, and rainwater. They assessed the quality of water by its appearance or taste: muddy, cloudy or glazed, smelly, or with debris or particles. The quality and safety of water are known to vary by season; especially with rains, water was deemed more dangerous because it could attract malaria mosquitoes and other infectious diseases, while during dry spells, water became difficult to access. There was a difference between water deemed suitable for washing and water deemed suitable for drinking. Liquid leftovers from washing and cooking were usually disposed of on the ground or into open sewage that animals and hens fed from.

In rural settings, humans and animals live closely. Observations in rural households in Burkina Faso showed that animals, especially poultry, roam freely but are often kept within the household at night to prevent them from being stolen. In group discussions with women, this was perceived to be a source of dirt, and the women would prefer that these animals remain outside. Given the level of infrastructure, it is likely that microbes and AMR genes can circulate between the domains identified by the One Health approach, humans, animals, and the environment, which creates a vicious cycle driving the use of antibiotics. More microbiological research is needed to determine if resistance genes are transmitted across these domains between human and animal species, but the lack of sanitation, and the potential flow of contaminated water, means that resistant infections are spreading. For example, Markkanen et al. (13) identified a worryingly high abundance of AMR genes in hospital water in the region.

### GAP objective 4: optimizing the use of antibiotics.

The WHO GAP guidance states that the increasing demand for antibiotics should be curbed to prevent the further evolution and spread of antimicrobial resistance and to ensure their continued use as a public good in the future. Our research suggests that rather than addressing a simple need to be curbed, antibiotics serve a purpose for people that should be understood rather than deemed an “irrational” or “unnecessary” problem that can be fixed. Denyer Willis and Chandler (14) describe antibiotics as a “quick fix” whereby they have come to play an important role by compensating for the lack of access to health care and health systems and managing the health issues caused by the missing sanitation infrastructures that would help prevent the spread of infections, for both animals and humans. The GAP suggests that the key to guaranteeing quality is optimizing the regulation of access to antibiotics for both humans and animals.

Our data show that antibiotics are used in support of fragile livelihoods that depend on antibiotics, driven by a lack of health care and the lack of diagnostic and surveillance infrastructure. The exact amount of antibiotics that are used at an aggregate level is unknown; national prescription data from West African countries do not exist, and the most representative data from Burkina Faso on antibiotic use suggest wide informal use: 40% of patients coming to the hospital had already taken antibiotics, 16% of whom had taken more than two different kinds of antibiotics (15). Of the antibiotics used, 40% were those that the WHO defines as being the most crucial to secure. Similarly, neither country has reliable data on the number and size of livestock businesses, many of which are unregistered small- to medium-scale holdings, with industrial and semi-industrial enterprises being concentrated in urban and periurban areas. The OIE has no data on veterinary services in either country. Anecdotal reporting for both countries states that there is a dearth of both public and private veterinary services in remote areas, with most commercial veterinary services being concentrated in urban areas. Relying on prescription data would inevitably generate a limited picture of the scale of use because of the complex routes of accessing antibiotics without a prescription.

In both countries, antibiotics are sold and prescribed by various frontline health care providers. Nurses prescribe antibiotics, and antibiotics can be bought directly from pharmacies and through informal markets without a prescription. Responsibility for how and why antibiotics are accessed was often deflected to others: doctors point to nurses, who point to pharmacists, who point to patient demands, etc. Collected using the “drug bag” method (16), our data from Burkina Faso show that households’ sources of antibiotics for treating humans include primary health care facilities (34%), informal sellers (27%), private sellers (29%), hospital drug stores (12%), community members (1.75%), and community health care workers (0.25%). To save money spent on drugs, it was common for people to buy antibiotics for a few days and then cease taking them when the patient had recovered. Here, hospitals are theoretically run by doctors, but in practice, nurses act as the frontline health care providers and are the main prescribers of antibiotics. Raising awareness about AMR would thus need to be targeted at nurses as well as informal sellers. In Benin, the informal sale of pharmaceuticals has been banned, but according to our findings, smuggling of drugs across the borders to the country continues. In a situation where sale is unregulated, informal drug selling offers a form of livelihood in places where there are few other employment options.

As we point out above in “Improving awareness of AMR through training and education,” awareness among frontline health care workers, doctors, nurses, veterinarians, and paraveterinarians about reducing the use of antibiotics was deemed to be present, yet antibiotics were used liberally. For example, our observational data and focus group discussions with animal breeders and farmers conferred that in animal husbandry, official dose regimens and recommendations were rarely followed as these would lead to considerable financial losses (17). While we did not find evidence of antibiotics being used for growth promotion, withdrawal periods after a course of antibiotics were rarely followed to ensure that farmers “break even” and to ensure continued food security for poor populations. Withdrawal periods would put fragile livelihoods at risk when breeders have no societal infrastructure to rely on. Also, nurses prioritized patient care: nurses sometimes skipped examinations knowing that patients could not afford them and prescribed antibiotics instead. The choice of antibiotic was made based on trial and error but often, to be on the safe side, with multiple antibiotics as suggested by the quote below. This situation could be improved by the availability of surveillance data and diagnostics.

Most often, depending on the type of case, one prescribes one antibiotic; currently, we have fallen into very wide use of ceftriaxone. If we see that things don’t improve, we can combine it with another antibiotic, which we think can act on the germ. If it is a very serious infection, go up to three antibiotics.

Moreover, a further problem in optimizing antibiotics concerned the quality of drugs available on the market. In Burkina Faso, a doctor was concerned about the quality of generic drugs.

Q: There is a depot (informal seller) in town; are these antibiotics the ones you would like to prescribe to your patients?

A: As long as there are specialties, you can get them. With generics, we don’t know where they come from…China, India, Nigeria. For generics, you don’t know if there is a minimum dose in them or it’s just flour or excipients that they put. So if you have the specialties, there is no problem.

Several interviewees in AMR policy discussions stated that quality control was lagging in terms of the regulation of pharmacies and various “unofficial” products on the market. The infrastructures needed to test “fake” or low-dose/low-strength drugs are lacking. The problem posed by substandard antibiotics is that even if people take the course as they should, if the efficacy of the drug is reduced due to age or handling, the dosage may be too low and can potentially lead to resistance or, if lacking the potency to effectively treat the patient, might trigger the use of another antibiotic. An interviewee in an international AMR policy organization argued that international organizations sometimes donated drugs that were either close to the expiration date or had already expired. He stated that while the authorities knew better than to distribute them across their drug dispensaries due to the potential risk of driving resistance, they did not have the means to dispose of them, which risked increasing environmental contamination with antibiotic residues. A microbiologist in Burkina Faso described the following challenges in quality control:

We don’t have strong capacity to do a good quality control…We don’t do the postmarketing control. Normally, you have three phases of control: preshipment control, control before they enter country—this is in the national lab—and, when the product is on the field, postmarketing control. But we don’t have the money to make a postmarketing control. When the product enters the country, it’s finished: is it good, is not good? (laughs) We don’t have the means to test it. Once we made a study, we went in the field, and we collected some antibiotics and evaluated them. In Burkina Faso and the (known) quality antibiotic represent between 30, 35%. The big problem for AMR is the regulation and the capacity to control the quality of antibiotics, but we don’t have this capacity.

Underlying the challenges in implementing the above-described objectives are finances, which we describe below.

### GAP objective 5: making an economic case for alternatives.

The WHO GAP calls for investment to develop new alternatives to antibiotics as well as improving the capacities for utilizing evidence-based interventions, diagnostic tools, and vaccines. It also states that the broader socioeconomic impacts of AMR should be considered, in terms of preventing infections but also planning new interventions, i.e., in terms of both the implications for taking no action toward curbing AMR and the financial risks of shifting away from the use of antibiotics.

Economic aspects of AMR are incredibly diverse and are arguably at the core of what is at stake when countries like Burkina Faso and Benin are developing their AMR resilience plans. The examples that we present here are among but a few of the relevant issues. Our selected examples point to the compounding economic limitations that poor countries face in setting up their policies, practices, and infrastructures relevant to AMR.

Our interlocutors in West Africa described that funding for various activities to control AMR, such as creating and establishing national action plans, strengthening policy networks, enforcing AMR stewardship, scaling up surveillance, training, and performing AMR-related research, etc., tended to come in short-term projects. International funding for fighting AMR had provided momentum for the implementation of AMR-related interventions in both countries, but the situation remains fraught due to unstable funding streams that must be applied for via international AMR-related funding calls. This means that they put their energies into applying for funds rather than designing implementation strategies, which skews the efforts: depending on the priorities set by the funders, bilateral development programs, international research funds, and philanthropic aid organizations, etc., different funds have specific foci, and as a result, some areas advance faster than others. Surveillance was described by several of our interlocutors as an area of international priority, but as indicated above in “Strengthening research and surveillance,” even that domain remains wanting.

An area where socioeconomic implications are and will be felt the most is in the livelihoods of farmers and those working in animal and bird husbandry and aquaculture. The section above reported the challenges for farmers and their reliance on antibiotics to avoid risking their livelihoods in cases of infection. At the same time, antibiotics are also a financial burden, exacerbated by the requirement to source veterinary supplies from approved overseas manufacturers, which leaves veterinarians and breeders vulnerable to fluctuations in currency exchange rates. To circumvent reliance on antibiotics, some smaller livestock businesses reported experimenting with local remedies known to have antibiotic properties, such as moringa, basil, neem, and vernonia, etc. These plants are common to the region, and farmers can gather the plants themselves. Such techniques are taught by private organizations giving training on sustainable farming (for example, Songhai Centre in Benin and Faso Elevage in Burkina Faso), as alternatives to chemical disinfectants and to treat or prevent bacterial infections. A breeder from Benin involved in the production of rabbits, roosters, guinea fowl, quail, and other types of poultry said,

A: I started using alternatives instead of going directly to the antibiotic. I use, for example, basil for the treatment of poultry. I grind it into drinking water, or I squeeze the leaves directly when I start to observe irregularities, which relate to the use of antibiotics.

Q: So you substitute antibiotics with certain leaves?

A: Yes, to reduce the cost of production because the purchase of veterinary products also increases the cost of production and reduces profits.

Q: And does it work?

A: Yes, it works.

Local researchers based in universities and civil society organizations were working to test and develop alternatives to antibiotics and to design probiotic feeds and products for use in farming that would support healthier microbiota and reduce conditions under which animal pathogens can spread (17). These endeavors remained localized and have not generated investment from international funders to support research or to scale up the products nationally or internationally, which would require verifications of safety and efficacy. Nonetheless, the use of plants was an important part of local efforts in reducing the use of antibiotics as described below by a Beninese breeder:

A: A nongovernmental organization gave us training last year. They gave us papers and plants that we can use to treat animals, by reducing not only the use of antibiotics but also the cost of productivity. Thanks to this training, I have reduced the use of antibiotics like Alfaceryl [a soluble antibiotic treatment specifically developed for animals manufactured by the Dutch pharmaceutical company Alfasan]. I do not know the name of the NGO, but I can contact them by their number.

Q: On what basis did this training take place? Did you notice that there was a problem?

A: I have a small herd. It was due to problems that we spoke to our (professional society) president who invited us for training; this training cost us 3,000 francs each. It’s true that we didn’t expect organic training. They asked us what problems we are having, and I told that the use of antibiotics is ruining me and I cannot reach my financial goals. They gave us plants that we can use: moringa, neem leaf, eucalyptus…These plants play the role of disinfectant and antibiotic; they fight against bacteria. They can do what the Alfaceryl can do.

## DISCUSSION

Our analysis of the present situation in terms of the implementation of the GAP in Benin and Burkina Faso shows that both countries have considerable difficulties in their ability to close the AMR policy implementation gap. Kirchhelle et al. (18) ask if a universal approach to AMR policy and antimicrobial stewardship is possible. In continuation, they ask, if yes, what hallmarks characterize “good” antibiotic policy? They provide three principles that describe a good policy: structural (recognizing different circumstances of antibiotic use), equitable (recognizing the unevenness in abilities to tackle AMR), and tracked (keeping abreast of effective interventions and ensuring that the two above-described hallmarks are fulfilled). Their critical analysis of ongoing attempts to strengthen stewardship programs and surveillance to address the problems of global objectives is essential, and we agree with the issues raised and the alternatives proposed. The value of having the GAP is its role as a roadmap to streamline efforts locally, and having a national action plan (NAP) in place presents attempts to localize them. Munkholm and Rubin (19) compared the textual similarities of the GAP and various NAPs and suggested that the degree of “parroting” of GAPs observable in the NAPs, rather than adaptation to the local context, speaks to the (in)ability of a country to introduce and implement global policies at the national level.

While having a genuine NAP in place is a crucial starting point for policy implementation, it does not guarantee that the policy is implemented. In their research on European policy governance structures for enacting AMR policies, Jon Pierre and Björn Rönnerstrand state that across Europe, there are considerable differences in how policies are instituted nationally. They argue that these differences in cultures of policy-making and governance explain some of the internal differences. Of key importance is the space and support given to scientific health policy-makers nationally to draft and implement policies (20). Of the 47 member states in the WHO African region, 15 have listed their NAPs on the WHO website (https://www.who.int/teams/surveillance-prevention-control-AMR/national-action-plan-monitoring-evaluation/library-of-national-action-plans), and evidence from elsewhere in Africa suggests that countries that have NAPs in place (21–23) have similarly been able to do so owing to the initiative, drive, and enthusiasm of national health policy-makers, government mandates to do so, and the availability of sustained international funding. This policy space is then essential for starting to close the gap between global and local policies.

In this paper, we have brought attention to the gaps between global policy and national-level implementation in two countries. We have described how existing structures were not sufficient to realize the various objectives suggested by the GAP. The multiple shortcomings are layered and compounded. For example, it is not possible to use surveillance data to inform veterinary or clinical decision-making if there are no surveillance data, and it is difficult to build surveillance systems if there is no diagnostic infrastructure, which one cannot establish if there is no system in place to deliver samples to laboratories, which will not happen if there is no awareness or expertise about the need to look for resistant infections, etc. This analysis has brought us to the remit of development and the overall spread of concerns that go over and beyond antibiotic use. A successful AMR response needs to be locally tailored, situated, and site specific. The work needed to implement policy objectives continues to fall on local policy-makers who must navigate and adapt these proposals to their contexts, often with very limited, short-term resources, and without local political support, they would find their efforts constrained.

This begs the question of whose role it is to invest in the development needed to implement NAPs and where to start. The World Bank has proposed that any development policy would need to have AMR-sensitive interventions written into it (24). Externally funded development programs come with the political-economic legacy of economic liberalization that has eroded the role of the state in those duties. However, the implementation of AMR-minded development programs cannot be outsourced to multilateral programs; AMR needs to be embedded in all the programs overseen by the state, including bilateral programs, to ensure that all of the gaps are closed.

Given that funds to enact AMR policy are limited in many low-income settings, where should local policy-makers begin? The challenge, as we have shown, is the need to prioritize efforts and find out what would be most efficient in a given context, with the added difficulty that the actions required are interdependent. Given that the structures within which bacteria and AMR genes spread are highly localized and result from local circumstances, there cannot be a universal silver bullet. Interventions need to be responsive to local needs, hot spots, driving factors, and circumstances that give rise to the use of antibiotics, which are always and inevitably contingent and situated. In one site, the key problem might be a drug development factory that leaks its effluent water to a nearby river. In another location, it may be an unregistered village that has no formally registered right to a health care center, and the nearest primary health care unit is a long walk away, which drives up informal antibiotic use. In a third site, breeders may have no social insurance in cases of financial loss owing to a disease, which leads them to not comply with withdrawal periods for selling their eggs, etc. All of these examples would require different kinds of intervention. Identifying which domains of the NAP and which sites would be most efficacious in a given setting requires interdisciplinary and policy research. Social sciences can support local policy-makers in identifying key priorities and the practices, reasons, causes, and/or factors that drive hot spots as well as identifying potential places where interventions could be made in culturally sensitive and meaningful ways. Local political will and access to sustained resources are crucial in the process of prioritization.

### Conclusion.

In this paper, we have explored the feasibility and challenges of implementing the global AMR action plan in two of the world’s poorest countries, Benin and Burkina Faso. We show that preventing AMR spread and promoting controlled antibiotic use depend on addressing various stacked lacks that can broadly be defined as development. Such broad sweeping needs beg the question of where to start. We suggest that local research-based priority setting is necessary to address local needs.

## MATERIALS AND METHODS

This study formed part of an international multidisciplinary project consortium. The consortium is composed of medical and environmental microbiologists, molecular biologists, and social scientists who monitor the flow of antimicrobial resistance among people, nonhuman animals, and environments. The research team consisted of social scientists and microbiologists from both Europe and West Africa. The material for this study concerns the project’s social science component.

The study was conducted in accordance with relevant guidelines and regulations for conducting qualitative research. Ethical clearance was gained from the Comité National d’Ethique por la Recherche en Santé (Benin) and the Ministère de la Santé, Ministère de l’Enseignement Supérieur, de la Recherche Scientifique, et de l’Innovation, Comité d’Ethique pour la Recherche en Santé (Burkina Faso).

Data were collected on themes of clinical health care, livestock breeding, and water, hygiene, and sanitation (WASH). Data gathering took place between 2019 and 2020, applying primarily qualitative and ethnographic approaches but also including household surveys of WASH and health management practices in rural villages in the Savalou Commune of Benin and the Nanoro District of Burkina Faso, urban households in the cities of Calavi (Benin) and Ouagadougou (Burkina Faso), and urban gardeners in Ouagadougou. This study uses interview and focus group quotations to illustrate findings supported and informed by the survey data. Fieldwork in Burkina Faso during 2020 was halted early due to the global coronavirus disease 2019 pandemic.

We conducted a total of 80 interviews with livestock breeders, clinicians, laboratory staff, urban gardeners, and urban residents.

Table 1 breaks down interviewee information by occupation and country.

**TABLE 1:** Interviewee information by occupation and country

Interviewee occupation	No. of interviewees	
	Benin	Burkina Faso
Surveillance and laboratory staff	3	6
Officials and ministry representatives	7	6
Nurses	1	0
Doctors	7	3
Livestock breeders	4	3
Veterinary scholars	1	0
Veterinary pharmacists	2	1
Paraveterinarians	2	2
Private breeder training organizations	2	1
Households	20	3
Urban gardeners	0	7

In the livestock sector, we interviewed seven breeders, three nongovernmental training agencies, a professor of veterinary science, three veterinary pharmacists, and three government paraveterinarians about their challenges in implementing antimicrobial stewardship. All interviewees were male. Of the breeders interviewed, most had either trained as farm laborers before establishing their own farms, received vocational training overseas, or both. One claimed to be self-trained. Paraveterinarians had received a diploma or bachelor’s certificate in animal production or agronomy science, while the ministry agent was a qualified veterinary doctor. We interviewed nine surveillance or laboratory researchers (two female and seven male, all with higher education), two researchers from clinical laboratories (one female and one male), and one researcher from a veterinary laboratory (male). We interviewed 1 nurse (female) and 10 doctors (1 female doctor, 6 male doctors, 1 male surgeon, and 2 French Canadian pediatricians [1 male and 1 female]). We interviewed 13 ministry officials and local representatives of international organizations responsible for implementing the GAP (4 female and 9 male, all with higher education). All but one of the professional interviewees were employed at the time of the interview. The one unemployed interviewee was a postdoc looking for funding but still conducting research actively.

Data on water supply, water consumption, and sanitation and hygiene practices were collected using three survey questionnaires: 23 from a large village in Central Benin; 50 urban households in Calavi, Benin; 30 households in Ouagadougou, Burkina Faso; and 25 urban gardeners in Ouagadougou, Burkina Faso. In addition, we conducted 9 interviews on the theme of WASH with urban gardeners and households in Ouagadougou and 20 households in Calavi, Benin. Respondents in the Calavi area were aged between 22 to 61 years and had diverse socioeconomic and educational backgrounds. Given their overall responsibility for WASH management in the household, 17 respondents were female, and 3 were male participants. In Ouagadougou, we interviewed seven urban gardeners and members of three households (one who also had a garden). The respondents were recruited from the same livelihood group; their socioeconomic backgrounds were similar. They were aged between 30 and 53 years, and eight of the respondents were male. More WASH data were collected using observations from villages in Central Benin and eight group discussions with 65 participants in total in three areas in rural Burkina Faso.

The surveys and interviews also included sections on self-reported health issues and treatment-seeking practices. A survey of 423 households employing the drug bag method was conducted in three rural communities in the Nanoro area of Burkina Faso to collect data on antibiotic recognition and use in humans and animals. The three communities were purposefully selected based on the Health and Demographic Surveillance System socioeconomic categorization (high, middle, or low). The survey investigated the use of antibiotics within the community. Since there are no previous studies or pilot studies that have estimated the prevalence of antibiotic use in the target population, a prevalence of 50% was used to calculate the sample size of the study. Thus, considering a confidence level of 95%, a precision of 5%, and 10% maximum missing data, a sample size of 423 households was used.

In addition, we observed three livestock agribusinesses, ranging from 5-day visits on two farms outside Ouagadougou in Burkina Faso combining intensive swine or poultry production with free-roaming cattle and small ruminants to a 3-week stay on a Beninese layer poultry farm. Breeding technicians were generally educated to the secondary level, although not all of them had gained their certificate. Some of our informants had participated in some form of vocational or technical training in animal production before being hired. Of those who participated in discussions, only two technicians could be categorized as lacking any kind of formal training or education.

The study also used data from nine focus groups with veterinarians and breeders in Benin and Burkina Faso. Topics included prescribing and diagnostic practices, disease treatment and prevention, hygiene and biosecurity practices, production practices, production and financial risk management strategies, and policy and regulation. In Benin, we conducted three focus group discussions with a total of 24 breeders, aged between 21 and 45 years. All of the breeders were male, which reflects the gendered characteristics of the sector in this country. Only three of the participants reported receiving academic training (to the level of a bachelor’s certificate) in agronomy or animal science. One participant reported receiving training as a paraveterinarian, 10 reported receiving some form of vocational training in animal production, and 9 reported receiving no training or described themselves as self-trained. One respondent did not list a response. In Burkina Faso, we had three focus groups with a total of 10 breeders, aged between 24 and 66 years. These groups included three females, aged between 26 and 43 years. Four of the respondents reported receiving training in technical management and/or business management. One respondent had received overseas training in France. Two reported being trained “on the job,” and two did not list a response. In Benin, we also conducted three focus groups with a total of 18 veterinarians, aged between 21 and 50 years. The groups included three female participants, aged between 21 and 26 years. With the exception of one respondent (who had received a certificate of primary education only), six of the veterinarian respondents had been educated to the level of a bachelor’s certificate, eight had been educated to the level of a master’s degree, and one had been trained to the doctoral level. We did not conduct any focus groups with veterinarians in Burkina Faso.

Interactions in the field, interviews, and focus groups took place either in English or in French, with local support for on-site interpretation if respondents spoke other languages as their preferred first language. Focus group and interview data were either digitally voice recorded or handwritten, while field observations were handwritten and typed up each day (or, in more remote areas, when availability of electricity allowed). Interview and focus group recordings in French were transcribed, translated using a digital translation service, and checked for accuracy. Surveys were collected by hand, and data were transferred to a spreadsheet.

Data were initially analyzed inductively to identify emergent themes. Based on a shared analytical framework, the data were then coded and analyzed using the Atlas.ti qualitative data analysis program by two coders independently (J. A. Cañada and A. Butcher), and the analysis was verified and cross-referenced by a third analyzer (S. Sariola). Data points for the manuscript were selected by the third analyzer (S. Sariola). Codes used for the study include policy, surveillance, awareness of AMR, antibiotic use, sanitation, and specific challenges in the implementation of these.
